# Medication delivery errors in outpatients with percutaneous endoscopic gastrostomy: effect on tube feeding replacement

**DOI:** 10.1038/s41598-023-48629-w

**Published:** 2023-12-08

**Authors:** David García González, Ana Martín-Suárez, Juan José Salvador Sánchez, Jesús Ángel Sánchez Serrano, M. Victoria Calvo

**Affiliations:** 1 Institute for Biomedical Research of Salamanca (IBSAL), Salamanca, Spain; 2 Departamento de Ciencias Farmacéuticas, Facultad de Farmacia, Universidad de Salamanca, Salamanca, Spain; 3 Emergency Service, University Hospital of Salamanca, Salamanca, Spain

**Keywords:** Endocrinology, Gastroenterology, Health care, Medical research, Risk factors

## Abstract

Patients with enteral access usually receive oral drugs via feeding tubes and correct drug administration remains a challenge. The aim of this study was to identify common medication delivery errors (MDEs) in outpatients with percutaneous endoscopic gastrostomy (PEG) and evaluate their association with the need for tube replacement due to deterioration or clogging. A 2-year retrospective study that comprised adult outpatients with a placed/replaced PEG tube and whose electronic medical record included home medication was carried out. Treatment with medication that should not be crushed and administered through an enteral feeding tube was considered an MDE. We included 269 patients and 213 MDEs (20% of oral prescriptions) were detected in 159. Ninety-two percent of the medications associated with MDEs could be substituted by appropriate formulations. Tube replacement due to obstruction was needed in 85 patients. MDEs were associated with increased risk for tube replacement (OR 2.17; 95% CI 1.10–4.27). Omeprazole enteric-coated capsules were associated with the greatest risk (OR 2.24; 95% CI 1.01–4.93). PEG outpatients are highly exposed to MDEs, leading to a significant increase in the odds of tube replacement, mainly when treated with omeprazole. The use of appropriate alternative therapies would prevent unnecessary adverse events.

## Introduction

Both inpatients and ambulatory care patients who cannot tolerate oral intake are dependent on enteral feeding^1^. Percutaneous endoscopic gastrostomy (PEG) is the most widely used procedure in long-term nutrition support. This gastric access device presents important advantages, such as easy placement, short hospital stays, favorable cost-effectiveness ratio, and safety. However, it might also lead to important and serious complications, especially if the appropriate care is not provided^2,3^. Complications following gastric access placement are reported in the literature to range between 8 and 30% for PEG^4^. One of the most commonly observed complications is tube obstruction, frequently associated with enteral feeding and medication administration errors^4–6^.

Patients with chronic enteral access usually receive oral drugs through the feeding tube, in which case oral dosage form modification may aid medication administration. Crushing tablets or opening capsules prior to dilution in water and delivery via the feeding tube are widely used practices; however, they can alter the efficacy and safety parameters of the drug, resulting in clinically significant consequences^7^. Crushing enteric-coated or controlled-release formulations allow the liberation and absorption of the drug in the stomach instead of the small intestine, which is the desired site. This can lead to irritation of the gastric mucosa, a decrease in the effect of the drug, and an increased risk of side effects such as feeding tube obstruction^8^. Obstructed feeding tubes often have to be replaced, resulting in increases in patient morbidity and expenses, especially in the case of PEG tubes, which frequently require endoscopic tube replacement.

A medication error is any preventable event that may cause or lead to inappropriate medication use or patient harm while the medication is under the control of the healthcare professional, patient, or consumer^9^. Accordingly, dosage formulation modification prior to administering the drug via feeding tubes constitutes a medication delivery error (MDE) associated with potential therapeutic risks and patient safety incidents^1,10^.

There are many reports on MDEs in patients receiving enteral tube feeding^1,10–13^. However, although PEG is commonly used, and these feeding tubes are prone to obstruction, there is a lack of awareness and knowledge about appropriate drug administration using this procedure. To improve patient care, stronger evidence bases to ensure the safety of drug delivery via PEG are required. The aims of this study were to identify common MDEs in outpatients with PEG and evaluate their association with the need for tube replacement due to obstruction or deterioration.

## Methods

### Study design and population

Retrospective cohort study carried out with outpatients attending the Endoscopy Unit of a large University Hospital from 1 February 2018 to 30 January 2020.

#### Inclusion criteria

Outpatients over 18 years of age treated in the Endoscopy Unit of the hospital and subjected to PEG tube placement or replacement interventions during the study period. Patients were only eligible if all possible complications could be assessed for at least 6 months from gastrostomy placement.

#### Exclusion criteria

Patients whose medical records did not include home medication information.

Patients were strict NPO receiving nutrition, hydration, and medications through the inserted PEG. Polyurethane 20 Fr feeding tubes were used in all patients. At our institution, this is the standard practice for patients with NPO orders who cannot take medication by mouth. This is a commonly used tube diameter to prevent clogging since it is more likely to occur when the gastrostomy tube is smaller than 18–20 Fr^14,15^. The nursing staff provided all the patients and caregivers with stoma care, tube feeding, and medication administration guidelines.

The British Association for Parenteral and Enteral Nutrition (BAPEN) and other scientific sources provide information on medicines that should not be crushed^16–18^. Based on these recommendations, the practice of crushing and administering the following medicines through the PEG was coded as the MDE:

- Modified/extended-release tablets or capsules and enteric-coated tablets. Altering the dosage form of these formulations affects the pharmacokinetic/pharmacodynamic profile of the drug. Moreover, these formulations tend to form clumps and are therefore more prone to blocking tubes.
- Soft gelatin capsules. They usually contain the drug in an oily viscous solution that tends to adhere to the wall of the tube and is resistant to flushing. Therefore, they are not suitable for administration via enteral feeding tubes.
- Cytotoxic and hormone medicines. These drugs present a great risk of occupational exposure.

Patients were classified into two groups: MDE and Non-MDE, depending on the presence or not of MDEs in the prescription, respectively. The major outcome of interest was based on feeding tube obstruction, and the association between the presence of MDE and obstruction that required tube replacement was evaluated.

### Data collection

Patient record reviews were conducted and the following data were collected: age, sex, diagnosis for PEG indication, PEG-related complications that required tube replacement, and number of times the tube had been replaced. Patient medication profiles were reviewed using electronic drug prescription records as the source of information. The oral medications administered via feeding tube from PEG placement to completion of the study were recorded. Subsequently, the exposure to MDE was coded and appropriate therapeutic alternatives for each of the drugs involved in the MDEs were assessed.

### Data analysis

Descriptive statistics were used to summarize the variables. Categorical variables were presented as frequency and percentage while continuous variables were reported as mean and standard deviation. A chi-squared test was used to assess the significance of the categorical data collected and an independent t-test was used to evaluate quantitative variables. The threshold for statistical significance was set at p < 0.05. Logistic regression was performed for the outcome variable of obstruction that required tube replacement and the following independent variables: age (< 75 or ≥ 75 years), sex, diagnosis, number of drugs administered via feeding tube, MDE exposure (yes or no), number of MDE (1 or > 1), formulation of the MDE medication (capsule or tablet), aspirin, omeprazole, pantoprazole, tamsulosin, and other drugs with MDE. In a multivariate backward stepwise regression analysis, certain variables were removed according to their statistical significance. The final model with odds ratios (OR) and 95% confidence intervals (CI) is displayed. All the analyses were conducted using IBM SPSS Statistics version 25.

### Ethical approval

This observational study was conducted in accordance with the Declaration of Helsinki and national and institutional standards. The research protocol was reviewed and approved by the drug research ethics committee (CEIm) of the health area of Salamanca, Spain (study ID: PI 2021-05-793). This is a retrospective study and had no impact on patient clinical management, for this reason, the committee approved the waiver of informed consent from patients.

## Results

During the study period, PEG tube interventions were performed in 402 patients, of whom 269 met the inclusion criteria. The main reason for exclusion was the lack of electronic prescription records in the case of deceased patients (n = 116, mean age (SD): 79.8 (13.8) years). The clinical characteristics of the patients included in the study are shown in Table 1. The medication reviews revealed 213 MDEs (20% of total oral prescriptions), corresponding to 159 (59%) patients, yielding an average of 1.3 MDEs per patient. Table 2 shows the drugs involved in the MDEs detected and the possible therapeutic alternatives.

**Table 1:** Baseline characteristics of patients included in the study.

	MDE group	Non-MDE group	*P*-value
Patients, n (%)	159 (59.1)	110 (40.9)	NA
Female	94 (59.1)	59 (53.6)	0.37
Age (years), mean (SD)	80.1 (10.3)	76.5 (15.2)	0.02
Oral medications administered via PEG, n/patient, mean (SD)	4.04 (1.3)	3.65 (1.5)	0.02
Indication for gastrostomy, n (%)			
Neurologic disease	128 (80.5)	71 (64.5)	< 0.01
Neoplastic disease	23 (14.5)	34 (30.9)	
Other diseases	8 (5)	5 (4.6)	

*MDE* medication delivery error, *NA* not applicable, *PEG* percutaneous endoscopic gastrostomy.

**Table 2:** Prescribed medications that should not be administered through enteral feeding tubes and therapeutic alternatives.

Prescribed drug and dosage form	PC	TRO	Therapeutic alternative
	Patients (n)	Patients (n)	
Aspirin EC tablet	83	23	Non-coated tablet or effervescent tablet
Proton pump inhibitors			Esomeprazole MUPS tablet
Omeprazole EC capsule	35	18	
Pantoprazole EC tablet	18	5	
Tamsulosin MR tablet	19	8	Alfuzosin or doxazosin tablet
Iron			Drops, sachets, oral solution or dispersible tablet
MR tablet	12	4	
MR capsule	4	1	
Venlafaxine			Normal release tablet
MR capsule	11	5	
MR tablet	2	1	
Valproate EC tablet	7	3	Oral solution
Alprazolam MR tablet	5	4	Normal release tablet
Biperiden MR tablet	4	2	Normal release tablet
Mirabegron MR tablet	3	1	Medication within the same class
Quetiapine MR tablet	2	2	Normal release tablet
Clomethiazole soft capsule	2	2	Medication within the same class
Isosorbide mononitrate MR tablet	1	1	Normal release tablet
Ranolazine MR tablet	1	1	Medication within the same class
Carbidopa/levodopa MR tablet	1	0	Normal release tablet
Lithium MR tablet	1	0	Not available in our country
Vitamin A soft capsule	1	0	Not available in our country
Vitamin A + E soft capsule	1	0	Not available in our country

*EC* enteric coated, *MR* modified release, *MUPS* multiple unit pellet system, *PC* prescription count, *TRO* tube replacement due to obstruction or deterioration.

**Figure 1:**
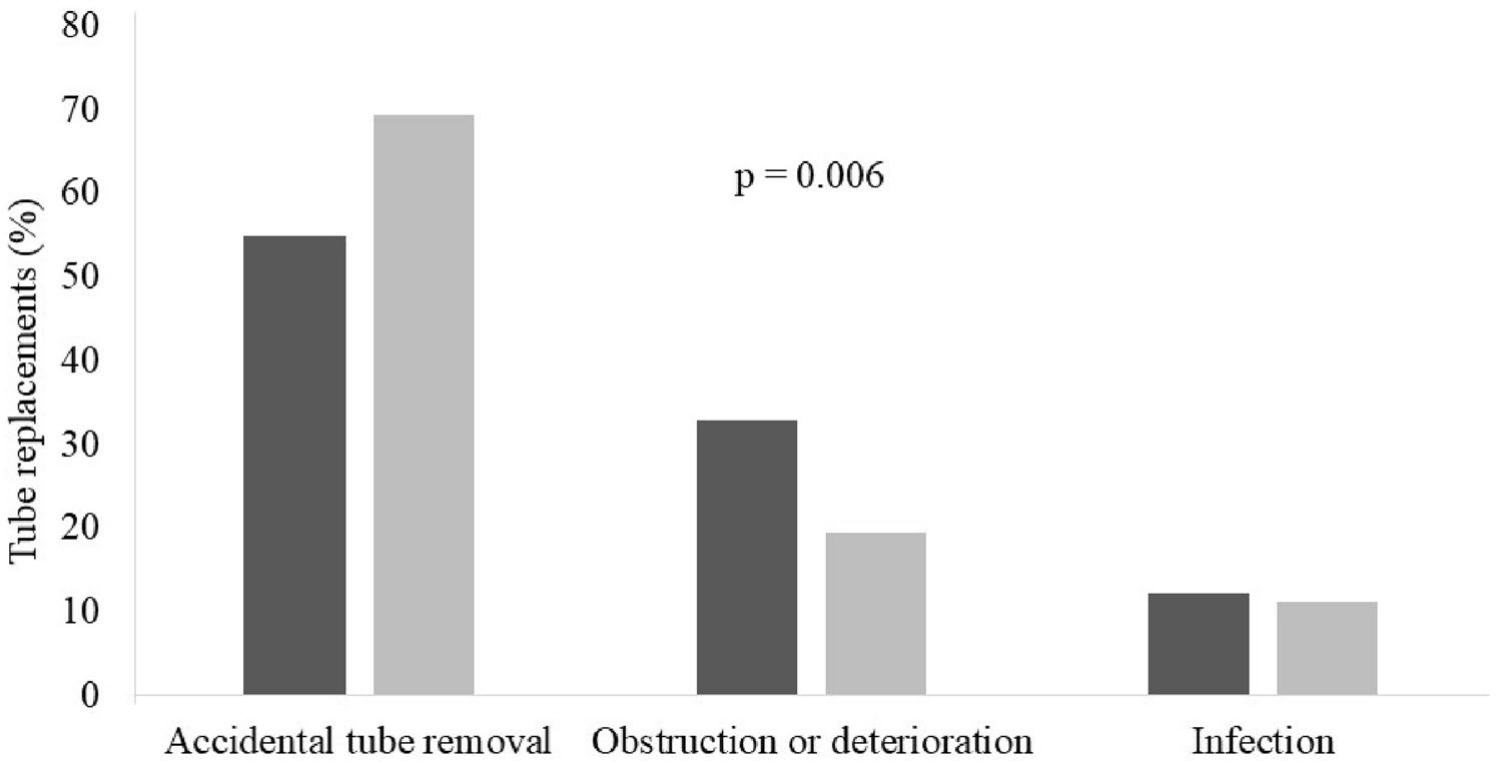
Tube replacements by different PEG complications in patients with (black filled square) and without (gray filled square) medication delivery errors.

PEG tubes were replaced in 196 (73%) of the 269 patients included in the study, with 431 PEG tube replacements: 262 and 169 in the MDE group and Non-MDE group, respectively. Figure 1 shows that accidental tube removal was the main reason for tube replacement, 55% in patients with MDE and 69% in patients without MDE. Tube obstruction was the second cause, with percentages of replacement because of this complication of 34% and 19% in the presence and absence of MDE, respectively (p = 0.006).

Eighty-five patients underwent at least one replacement due to tube obstruction and these events were recurrent in 24 of them. This PEG complication was significantly more frequent in patients with MDE, 36% (58 of 159) vs 25% (27 of 110) in Non-MDE patients (p = 0.04). Table 3 shows the association between MDEs and tube replacements. We analyzed the effect of MDE with omeprazole and aspirin as they were the most frequently prescribed drugs, finding that patients treated with omeprazole were the ones that experienced the highest number of tube replacements.

**Table 3:** Tube replacements due to obstruction or deterioration by MDE status.

	Non-MDE group	MDE group	Characteristics of MDE				
			1 MDE	> 1 MDE	Omeprazole	Aspirin	Other drugs with MDE
Patients, n	110	159	115	44	35	83	81
Recurrence of TRO, patients, n (%)							
Non-TRO	83 (75.4)	101 (63.5)	72 (62.6)	29 (65.9)	17 (48.6)	60 (72.3)	52 (64.2)
1 TRO	21 (19.1)	40 (25.2)	30 (26.1)	10 (22.7)	13 (37.1)	12 (14.5)	22 (27.2)
2 TRO	6 (5.5)	9 (5.7)	7 (6.1)	2 (4.5)	1 (2.9)	6 (7.2)	4 (4.9)
3 TRO	0 (0)	8 (5)	5 (4.3)	3 (6.8)	4 (11.4)	5 (6)	2 (2.5)
4 TRO	0 (0)	1 (0.6)	1 (0.9)	0 (0)	0 (0)	0 (0)	1 (1.2)
TRO per patient, mean (SD)	0.30 (0.57)	0.54 (0.86)	0.55 (0.86)	0.52 (0.87)	0.77 (0.97)	0.47 (0.87)	0.49 (0.81)

*MDE* medication delivery error, *SD* standard deviation, *TRO* tube replacement due to obstruction or deterioration.

Because age showed significant statistical differences between the groups, the results were analyzed according to this parameter. Seventy-one percent of patients (192 of 269) had an age ≥ 75 and 29% (77 of 269) were younger than 75. In older patients the following outcomes were found: 62% were exposed to MDEs, 28% presented obstruction and tube replacement, 15% received omeprazole, and 34% received aspirin. In patients aged < 75, these results were: 52%, 40%, 9%, and 23%, respectively.

Since an increasing incidence of the need for PEG tube replacement due to obstruction could be an important indicator of the effect of MDEs, we performed a logistic regression analysis to quantify the relative contribution of MDEs to the risk of tube obstruction. The results of the final logistic regression modeling are shown in Table 4. The following variables were excluded from the regression model: sex, diagnosis, number of administered drugs, number of MDEs, formulation of the MDE, and presence of MDE with pantoprazole, tamsulosin, or other drugs. The presence of MDEs was associated with a significant increase in the chance of obstruction requiring PEG tube replacement, and an age ≥ 75 slightly decreased this risk. Omeprazole and aspirin also modified the risk of obstruction.

**Table 4:** Final logistic regression model for obstruction that requires tube replacement as a dependent variable.

Independent variable	Coefficient	*p* value	Odds ratio	95% Confidence interval
MDE	0.775	0.025	2.172	1.104–4.272
Omeprazole	0.805	0.046	2.236	1.014–4.932
Aspirin	− 0.681	0.048	0.506	0.257–0.996
Age ≥ 75 years	− 0.662	0.025	0.516	0.290–0.919
Constant	− 0.702	0.014	0.496	

*MDE* medication delivery error.

## Discussion

PEG is most frequently reserved for patients requiring long-term nutrition support and it is generally indicated for neurological conditions and obstructive lesions of the upper gastrointestinal tract^19^. Accordingly, the majority of patients in our study cohort suffered from neurological diseases that affected swallowing (74%), and 21% of them had neoplastic diseases. In addition to enteral nutrition, these patients often need simultaneous drug therapy, which is usually administered through tube feeding. Crushing oral dosage forms prior to enteral medication administration is common in clinical practice; however, reported practices are inconsistent and the correct administration remains a challenge^20,21^.

Our study provides an analysis of the incidence of MDEs in patients with PEG in a homecare setting. Out of an average of 1049 oral medication prescriptions, we identified 213 MDEs. Fifty-nine percent of the patients were affected by at least one MDE, and 99% of them occurred with medications that should not be modified because of the risk of altering their pharmacological action^8,22,23^. Oral tablets and capsules that cannot be crushed or manipulated to be administered via feeding tubes must be transitioned to a different formulation. We have found that 96% of the medicines involved in MDEs could be substituted by liquid formulations or dispersible dosage forms (Table 2). In line with the literature, these results suggest that, although healthcare professionals are aware of the purpose of enteric or modified-release formulations, little is known about the risks associated with modifying oral dosage forms^13,24^. The medications that were most frequently used inappropriately were aspirin and proton-pump inhibitors (PPIs), which is consistent with the diagnoses of the patients included in the study^12,25,26^. In addition to the therapeutic problems caused by crushing solid oral forms, with mutagenic or teratogenic drugs this practice may result in harm to the nurse or caregiver administering the medication^8^. This is the case with vitamin A, which was delivered to two patients in our study without the necessary equipment for handling it^27^. Despite the regularity of these practices, there is scarce understanding of the risk of complications for the patients and healthcare professionals involved. Legal problems may arise as modification usually leads to off-label use of the drug where the prescriber and the person administering the medication are responsible for adverse events^4,17^.

In outpatients with PEG, our results suggest that tube obstruction and replacement risk were higher under exposure to MDEs. The results of the logistic regression analysis revealed four risk factors: the presence of MDE in the prescription, omeprazole-MDE, aspirin-MDE, and age. However, we did not register other known risk factors such as malignancy or the number of drugs administered via PEG, probably due to a lack of power. The setting where the care is provided, whether home or nursing home, could be another risk factor since the level of knowledge and training of the careers in charge of the administration of medicines and PEG care could be different. As this information did not appear in most of the electronic records, in this study it was not possible to analyze the effect of the type of caregiver. Nonetheless, other studies have also reported medication delivery errors as well as the need to improve safe medication management in both nursing homes and residential care environments^28,29^. Thus, there is no reason to expect significant differences in the results depending on whether the caregiver is a relative or a nurse.

The multivariate logistic regression confirmed that MDEs independently increased the risk of obstruction and tube replacement. These results are consistent with those of other studies where crushed medication has been found to contribute to enteral tube obstruction^12,19,21,25^. Crushing gastro-resistant or modified-release medications could increase damage and tube obstruction rates because of adhesion of the enteric coating or formulation excipients, among others, to the tube walls^11,19,30^.

The second tube obstruction risk factor concerned specific drugs. Patients receiving aspirin enteric-coated tablets were at lower risk of tube replacement caused by obstruction than those who received other drugs with MDE (OR 0.506; 95% CI 0.257–0.996). Aspirin is a highly soluble compound that is rapidly released from non-coated tablets; therefore, this characteristic could support our results^31^. As opposed to aspirin, omeprazole modified-release capsules are associated with an increased risk of obstruction and tube replacement (OR 2.236; 95% CI 1.014–4.932). PPIs are commonly regarded as problematic drugs to administer via enteral tube^12,21,26,32,33^. The enteric-coated dosage forms of these drugs should be crushed and dissolved in 8.4% sodium bicarbonate^8,33^. On the other hand, omeprazole administered as a suspension in sodium bicarbonate could not supply adequate drug for systemic absorption^33,34^. In fact, esomeprazole has been proposed as the PPI of choice for nasogastric tube feeding^33^.

Lastly, and surprisingly, being younger than 75 was associated with a higher risk for tube replacement, which is not consistent with other investigations^35^. Our results could be attributed to the fact that aspirin was more frequently prescribed among patients ≥ 75 years of age, with 65 out of 83 aspirin prescriptions.

Prevention is pivotal in trying to tackle the problem that MDEs pose. As a first step, it is imperative that the medication requirements of each patient be thoroughly reviewed and all nonessential drugs be deprescribed. The concept of deprescribing involves eliminating unnecessary and/or inappropriate medication and is associated with greater patient satisfaction, decreased costs and healthcare utilization, and elimination of the risk of adverse events^36^. PPIs are among the most widely prescribed drugs, with a high prevalence among healthy and community-dwelling older patients, although many prescriptions are baseless^37–40^. Indeed, PPIs could be considered potentially inappropriate for many older patients, offering deprescribing opportunities^41,42^.

Patients with PEG are very vulnerable and specifically in need of care and effective drug therapy. In-home enteral feeding drug selection and administration can cause problems for patients and caregivers. However, caregivers of PEG patients in home settings usually lack sufficient discharge training and support and may have difficulties in administering medications via tube feeding, which can affect therapeutic results and patient safety. In fact, they should have appropriate medication formulations readily available rather than having to modify the available products^32^.

The MDEs detected in these PEG patients result in an unnecessary increase in adverse events and expenses for the healthcare system. In patients who are at home or in long-term care settings, a clogged tube may require a visit to the emergency department, increasing the burden of patient care in addition to replacement costs. It is our duty to provide the best healthcare and minimize these events to improve patient safety, extend the average life of PEG tubes, and reduce costs^7^. Enteral medication for tube-fed patients can be safe and effective when appropriately selected drugs are used together with best practices for their preparation and administration^14^.

The ESPEN guidelines for home enteral nutrition point out that the process should be standardized and coordinated by a multidisciplinary nutrition support team as this increases the quality of the process and reduces complication rates, thus making a significant contribution to improving patients’ quality of life and to the cost-effectiveness of the nutrition provided^43^. Nevertheless, there are insufficient data to accurately determine the degree of effectiveness of this type of intervention. These guidelines recommend the involvement of a pharmacist to provide prescribers, patients, and carers with the appropriate information regarding the administration of medicines via tube feeding. Indeed, there are several reports of positive results achieved by clinical pharmacists^13,28^. Collaborative approaches involving a multidisciplinary team to promote the correct administration of medications through enteral feeding tubes have been published, reporting substantial improvements in reducing obstruction events and the proportion of medication errors^10,11,13^. Efforts to raise awareness of the problem, the development of interdisciplinary best practice guidelines, and investing in caregiver training could improve the practice of drug delivery and enteral tube feeding.

Although our study contributes novel information on MDEs in PEG patients, there are several limitations. In our country, certain drugs such as laxatives are available over the counter, and their use may be underestimated in this cohort. Moreover, there was a lack of electronic prescription data for a significant number of patients, which could contribute to the misestimation of MDEs. Another possible limitation is the fact that we conducted this evaluation with patients attended at a tertiary hospital, which may restrict the generalizability of our results. However, the purpose of this study was to compare PEG tube obstruction and replacement rates based on MDEs in prescription and administration among patients at our hospital, and not to report absolute statistics. In any case, it is clear from the literature that comparable difficulties with the administration of drugs through enteral feeding tubes are found in many different hospitals. Finally, this is a retrospective study where all the data were obtained from information stored in electronic medical records.

Actual clinical outcomes as a result of MDEs were beyond the scope of this study. Nevertheless, a decreased need for PEG-tube replacement entails lower rates of medication toxicity and reduces the need for patient mobility. There is a growing number of patients receiving home enteral nutrition via gastrostomy, but few studies have evaluated medication administration by such route in community care patients.

In conclusion, overall exposure to MDEs is relatively high in outpatients with PEG, being associated with a significant increase in the odds of needing feeding tube replacement. Omeprazole capsules are the highest-risk medication, so our results do not support its administration through enteral feeding tubes. This study could raise awareness and improve knowledge regarding appropriate medication via PEG in patients in home settings. Future prospective studies could be needed to establish a causal relationship between MDEs and the need for tube replacement.

## Data Availability

The data sets generated and/or analyzed during the current study are not publicly available due to the data confidentiality requirements of the ethics committee, but they are available from the corresponding author upon reasonable request and approval from the ethics committee.

## References

[1] Williams NT (2008). Medication administration through enteral feeding tubes. Am. J. Health Syst. Pharm..

[2] Rahnemai-Azar AA, Rahnemaiazar AA, Naghshizadian R, Kurtz A, Farkas DT (2014). Percutaneous endoscopic gastrostomy: Indications, technique, complications and management. World J. Gastroenterol..

[3] Sezer RE, Ozdemir Koken Z, Senol Celik S (2020). Home percutaneous endoscopic gastrostomy feeding: Difficulties and needs of caregivers, qualitative study. J. Parenter. Enteral Nutr..

[4] Kurien M, Penny H, Sanders DS (2015). Impact of direct drug delivery via gastric access devices. Expert. Opin. Drug Deliv..

[5] Blumenstein I, Shastri YM, Stein J (2014). Gastroenteric tube feeding: Techniques, problems and solutions. World J. Gastroenterol..

[6] Garrison CM (2018). Enteral feeding tube clogging: What are the causes and what are the answers? A bench top analysis. Nutr. Clin. Pract..

[7] Boullata JI (2017). ASPEN safe practices for enteral nutrition therapy. J. Parenter. Enteral Nutr..

[8] Bandy KS, Albrecht S, Parag B, McClave SA (2019). Practices involved in the enteral delivery of drugs. Curr. Nutr. Rep..

[9] National Coordinating Council for Medication Error Reporting and Prevention. *Medication Error Taxonomy*. http://www.nccmerp.org/about-medication-errors (1998).

[10] Pereira RA (2020). Quality improvement programme reduces errors in oral medication preparation and administration through feeding tubes. BMJ Open Qual..

[11] Van den Bemt PM (2006). Quality improvement of oral medication administration in patients with enteral feeding tubes. Qual. Saf. Health Care.

[12] Sohrevardi SM (2017). Medication errors in patients with enteral feeding tubes in the intensive care unit. J. Res. Pharm. Pract..

[13] Cavagna P (2022). Assessment of good practice guidelines for administration of drugs via feeding tubes by a clinical pharmacist in the Intensive Care Unit. Crit. Care Nurse.

[14] Boullata JI (2021). Enteral medication for the tube-fed patient: Making this route safe and effective. Nutr. Clin. Pract..

[15] Wensel TM (2009). Administration of proton pump inhibitors in patients requiring enteral nutrition. P. T..

[16] British Association for Parenteral and Enteral Nutrition (BAPEN). In *Medications. *https://www.bapen.org.uk/nutrition-support/enteral-nutrition/medications (BAPEN, 2017).

[17] White R, Bradnam V (2015). Handbook of Drug Administration Via Enteral Feeding Tubes.

[18] Wright, D. *et al*. Medication management of patients with nasogastric (NG), percutaneous gastrostomy (PEG), or other enteral feeding tubes. In *Guidelines. *https://www.guidelines.co.uk (MGP Ltd, 2019).

[19] Arvanitakis M (2021). Endoscopic management of enteral tubes in adult patients—Part 1: Definitions and indications. European Society of Gastrointestinal Endoscopy (ESGE) guideline. Endoscopy.

[20] Phillips NM, Endacott R (2011). Medication administration via enteral tubes: A survey of nurses' practices. J. Adv. Nurs..

[21] Tillott H (2020). Survey of nurses' knowledge and practice regarding medication administration using enteral tubes. J. Clin. Nurs..

[22] Mercovich N, Kyle GJ, Naunton M (2014). Safe to crush? A pilot study into solid dosage form modification in aged care. Australas. J. Ageing..

[23] Caussin M (2012). L'écrasement des médicaments en gériatrie: Une pratique «artisanale» avec de fréquentes erreurs qui nécessitait des recommandations (Crushing drugs in geriatric units: A "handicraft" practice with frequent errors which imposed recommendations). Rev. Med. Interne.

[24] Demirkan K, Bayraktar-Ekincioglu A, Gulhan-Halil M, Abbasoglu O (2017). Assessment of drug administration via feeding tube and the knowledge of health-care professionals in a university hospital. Eur. J. Clin. Nutr..

[25] Borges JLA (2020). Causes of nasoenteral tube obstruction in tertiary hospital patients. Eur. J. Clin. Nutr..

[26] Heineck I, Bueno D, Heydrich J (2009). Study on the use of drugs in patients with enteral feeding tubes. Pharm. World Sci..

[27] Hodson L (2023). Managing Hazardous Drug Exposures: Information for Healthcare Settings. Publication No. 2023–130.

[28] McDerby N (2019). The effect of a residential care pharmacist on medication administration practices in aged care: A controlled trial. J. Clin. Pharm. Ther..

[29] Dilles T, Elseviers MM, Van Rompaey B, Van Bortel LM, Stichele RR (2011). Barriers for nurses to safe medication management in nursing homes. J. Nurs. Scholarsh..

[30] Seifert CF, Johnston BA (2005). A nationwide survey of long-term care facilities to determine the characteristics of medication administration through enteral feeding catheters. Nutr. Clin. Pract..

[31] Karkossa F, Klein S (2017). Assessing the influence of media composition and ionic strength on drug release from commercial immediate-release and enteric-coated aspirin tablets. J. Pharm. Pharmacol..

[32] Alsaeed D, Furniss D, Blandford A, Smith F, Orlu M (2018). Carers' experiences of home enteral feeding: A survey exploring medicines administration challenges and strategies. J. Clin. Pharm. Ther..

[33] Messaouik D, Sautou-Miranda V, Bagel-Boithias S, Chopineau J (2005). Comparative study and optimization of the administration mode of three proton pump inhibitors by nasogastric tube. Int. J. Pharm..

[34] Sharma VK, Peyton B, Spears T, Raufman JP, Howden CW (2000). Oral pharmacokinetics of omeprazole and lansoprazole after single and repeated doses as intact capsules or as suspensions in sodium bicarbonate. Aliment. Pharmacol. Ther..

[35] Pih GY (2018). Risk factors for complications and mortality of percutaneous endoscopic gastrostomy insertion. BMC Gastroenterol..

[36] Scott IA (2015). Reducing inappropriate polypharmacy: The process of deprescribing. JAMA Intern. Med..

[37] Triantafylidis LK, Hawley CE, Perry LP, Paik JM (2018). The role of deprescribing in older adults with chronic kidney disease. Drugs Aging.

[38] Lockery JE (2020). Prescription medication use in older adults without major cardiovascular disease enrolled in the aspirin in reducing events in the elderly (ASPREE) clinical trial. Pharmacotherapy.

[39] Ali O, Poole R, Okon M, Maunick S, Troy E (2019). Irrational use of proton pump inhibitors in general practise. Ir. J. Med. Sci..

[40] Liu Y, Zhu X, Li R, Zhang J, Zhang F (2020). Proton pump inhibitor utilization and potentially inappropriate prescribing analysis: Insights from a single-centred retrospective study. BMJ Open.

[41] American Geriatrics Society (2019). Beers Criteria® for potentially inappropriate medication use in older adults. J. Am. Geriatr. Soc..

[42] Farrell B, Lass E, Moayyedi P, Ward D, Thompson W (2022). Reduce unnecessary use of proton pump inhibitors. BMJ.

[43] Bischoff SC (2022). ESPEN practical guideline: Home enteral nutrition. Clin. Nutr..

